# Melatonin Decreases Acute Inflammatory Response to Neural Probe Insertion

**DOI:** 10.3390/antiox11081628

**Published:** 2022-08-22

**Authors:** Daniela D. Krahe, Kevin M. Woeppel, Qianru Yang, Neetu Kushwah, Xinyan Tracy Cui

**Affiliations:** 1 Department of Bioengineering, University of Pittsburgh, Pittsburgh, PA 15260, USA; 2 Center for the Neural Basis of Cognition, Pittsburgh, PA 15213, USA; 3 McGowan Institute for Regenerative Medicine, Pittsburgh, PA 15219, USA

**Keywords:** melatonin, glial scar, insertion trauma, anti-inflammatory, antioxidant

## Abstract

Neural electrode insertion trauma impedes the recording and stimulation capabilities of numerous diagnostic and treatment avenues. Implantation leads to the activation of inflammatory markers and cell types, which is detrimental to neural tissue health and recording capabilities. Oxidative stress and inflammation at the implant site have been shown to decrease with chronic administration of antioxidant melatonin at week 16, but its effects on the acute landscape have not been studied. To assess the effect of melatonin administration in the acute phase, specifically the first week post-implantation, we utilized histological and q-PCR methods to quantify cellular and molecular indicators of inflammation and oxidative stress in the tissue surrounding implanted probes in C57BL/6 mice as well as two-photon microscopy to track the microglial responses to the probes in real-time in transgenic mice expressing GFP with CX3CR1 promotor. Histological results indicate that melatonin effectively maintained neuron density surrounding the electrode, inhibited accumulation and activation of microglia and astrocytes, and reduced oxidative tissue damage. The expression of the pro-inflammatory cytokines, TNF-α and IL-6, were significantly reduced in melatonin-treated animals. Additionally, microglial encapsulation of the implant surface was inhibited by melatonin as compared to control animals following implantation. Our results combined with previous research suggest that melatonin is a particularly suitable drug for modulating inflammatory activity around neural electrode implants both acutely and chronically, translating to more stable and reliable interfaces.

## 1. Introduction

Neural interfaces are paramount to countless research and clinical applications. These devices intimately interface with the central and peripheral nervous systems where they record the electrical communications between cells or drive activity by stimulation. Recording and stimulation are two principal tools that neural electrodes facilitate for the study of neurological function and structure [1,2,3,4]. These devices have been used in important research involving the diagnosis and treatment of neurological events such as Parkinson’s, psychiatric disorders, and stroke [5,6,7,8]. Especially remarkable is the recent development of neural interfaces that allow control of machinery, such as prosthetics through brain-machine interfacing [9,10]. Unfortunately, these avenues have been impeded by the long-term decrease in recording quality and ultimately the chronic failure of these devices. A major driver of long-term electrode failure is the host’s inflammatory responses to the implanted device, and both acute and chronic inflammation following the insertion of the electrodes prevent these devices from reaching their full potential. This inflammation results in activation of host glial cells, generation of reactive oxygen species (ROS), and loss of neurons surrounding the implant [11,12,13,14,15].

Drug delivery has been heavily employed to control and modulate the inflammatory tissue reactions which occur after electrode implantation. Among the most promising candidates are anti-inflammatory compounds such as resveratrol, dexamethasone, and melatonin [16,17,18,19]. Of these compounds, melatonin has shown particularly promising results. Melatonin (MT) is a naturally occurring hormone and has been shown in many studies to possess anti-inflammatory, antioxidative, and anti-apoptotic qualities [20,21,22,23,24,25,26,27]. Known widely for its participation in circadian rhythm during sleep-wake cycles, MT’s immune-related properties have also been noted in the past few decades, making it an attractive candidate for many drug-focused therapies [25,28,29]. MT acts via receptor-mediated suppression of the NF-KB pathway, thus reducing the inflammatory cytokines produced during local injury and other downstream inflammatory products like TNF-α and IL-6 [16,21,28,29,30,31]. In addition to this, MT also has a direct and potent ability to scavenge free radicals and reactive oxygen species [32,33,34,35].

MT’s antioxidant and anti-inflammatory properties are effective at mitigating the effects of foreign body response over time. In a study investigating the effects of acute and chronic MT administration, chronic administration of MT (daily injection for 16 weeks) resulted in higher quality recording for 16 weeks [36]. Interestingly, stopping melatonin treatment after two weeks resulted in a rapid decline in recording performance approaching that of control animals in just a few weeks.

Endpoint histological studies at 16 weeks have demonstrated that chronic MT injection can reduce neural inflammation and oxidative stress in the vicinity of neural electrode implantation [36]. However, a new analysis of MT’s acute effect is necessary to understand its effects in the early stages of implantation, where tissue reactions are dominated by insertion trauma and acute inflammation rather than just the chronic presence of the electrode. In addition to immunohistological analyses, polymerase chain reaction (PCR) may contribute to a more precise observation of the acute inflammatory pathways modulated by melatonin. Finally, in vivo two-photon microscopy (TPM) was used to monitor how MT treatment influences the inflammatory responses to intracortical neural electrodes in CX3CR1-GFP mice for seven days. As the first step toward a more comprehensive investigation, this study aims to identify the occurrence as well as the mechanism of these events during the first week of implantation.

## 2. Materials and Methods

Experiments were designed to determine the effects of melatonin treatment during the acute neural electrode implantation period. The host tissue response in control (saline-treated) animals and melatonin (MT) treated animals after one week of implantation of neural electrodes was examined via TPM and post-mortem immunohistochemical and RNA expression analysis.

### 2.1. Histological Study

#### 2.1.1. Surgical Procedures

Six C57BL/6 male mice were randomly split into control and experimental groups (*n* = 3). Throughout the study, animal experiment and housing were approved by and kept in accordance with University of Pittsburgh Institutional Animal Care and Use Committee standards. Mice from all groups received daily 100 µL intraperitoneal (IP) injections three days preceding and seven days following probe implantation. Animals allocated to the control group received saline, while MT-treated animals received MT (MP Biomedicals, Solon, OH, USA) in saline at 30 mg/kg, with minimal DMSO added to increase solubility.

Surgeries were performed as described in previous experiments [36]. Animals were anesthetized using an isoflurane vaporizer (2% in oxygen for induction, 1.5% for remainder of procedure), and confirmed to be unconscious with the toe pinch method. After induction, each animal was mounted to a stereotactic frame (Kopf Instruments, Tujunga, CA, USA) and warmed with a hot water pad (TP1500, Adroit Medical Systems, Loudon, TN, USA) at 37 deg C. After shaving and sterilization of the implantation region (iodine and ethanol), an incision was made to expose the lambda and bregma lines of the skull. In reference to the coronal suture, bone screw sites were made bilaterally 1 mm anterior to lambda and 1.5 mm distal from the midline using a surgical drill (0.007 drill bit, Fine Science Tools, Inc., Foster City, CA, USA). Saline washes on the surface of the skull were performed to protect the animal and dissipate the heat caused by drilling. Two bone screws (Stainless Steel; shaft diameter: 1.17 mm, length: 4.7 mm; Fine Science Tools, Inc., Foster City, CA, USA) were screwed into the newly formed holes, with careful attention to not pierce the brain. Two electrode holes were drilled 2 mm posterior to lambda and 2 mm from the midline, and electrodes (NeuroNexus) were inserted and sealed via Kwik-Cast Sealant (World Precision Instruments, Sarasota, FL, USA). Dummy probes were utilized in this study as opposed to functional probes due to prohibitive costs and the lack of necessity for the selected experiments. Recording data has been collected previously and can be found in a previous work [36]. Dental cement (Pentron Clinical, Orange, CA, USA) was then generously delivered and cured with UV light around all four sites to anchor the probes in place to form a protective head cap. Incision sites were sutured and treated with a topical antibiotic, and animals received an intraperitoneal injection of 5 mg/kg Ketofen (100 mg/mL, Zoetis Inc, Kalamazoo, MI, USA). Mice were placed back in their respective cages on top of a warming pad while they regained consciousness. Ketofen injections (5 mg/kg) continued daily for three days after surgery for pain management.

At 1 week, animals were given an overdose of ketamine/xylazine (80−100 mg/kg and 5−10 mg/kg, respectively), which was confirmed by absent toe/tail pinch reflex. An incision was made to expose the diaphragm, which was cut away to expose the chest cavity. Mice were perfused with 100 mL phosphate-buffered saline (PBS) followed by 100 mL 4% paraformaldehyde (PFA) in PBS. The bottom of the skull was removed to expose the brain and the skull was post-fixed in 4% PFA at 4 deg C for 4−6 h. Brains were sucrose protected, frozen, and cryosectioned at 10μm thick slices.

#### 2.1.2. Immunohistochemistry (IHC)

Tissue sections containing the implant injury were stained immunohistochemically. Staining was performed by rehydrating the slices in citrate buffer and then blocking with 10% goat serum, following which the brain slices were treated with 0.1% Triton-x for 45 min. Staining of the brains was performed in groups consisting of NeuN (Millipore mouse 1:250), NF200 (Abcam rabbit 1:500), Iba-1 (Millipore mouse 1:500), GFAP (DAKO rabbit 1:500), tomato lectin (Vector Labs mouse 1:250), 4HNE (Oxisresearch mouse 1:200), and Caspase-3 (Cell Signaling mouse 1:500). The chosen stains were intended to detect the presence of reactive astrocytes (GFAP), neuronal axons (NF200) and density (NeuN), vasculature and microglia (tomato lectin), microglia (Iba-1), lipid peroxidation (4HNE), and apoptosis (Casp-3) surrounding the implant sites. Comparisons were drawn between control and MT-treated groups based on the relative intensity of these antibodies, especially glial activation and scarring, oxidative stress, and neuron availability surrounding the implanted electrodes.

#### 2.1.3. IHC Image Analysis

Images of slices were obtained using confocal fluorescent microscopy (Olympus Fluoview 1000) to evaluate the differences in tissue composition surrounding the implant sites. Images were analyzed in a custom Matlab script. Bins of 10 µm were created concentrically around the probe implant and the intensity of the stain or number of cells labeled per bin was measured. Intensities and counts were scaled to control regions at the corners of the images. Background intensity was calculated from the corners of the image (20% of the total image area) by removing pixels greater than 1 standard deviation (STD) above the mean and calculating the mean and variance of the remaining pixels. All pixels greater than 1STD above the mean background intensity were used for analysis. Manual blinded counts within masked bins were performed to obtain the data for NeuN and Caspase-3. In the NF200, 4HNE, and tomato lectin analysis, *n* = 14 images were analyzed and averaged for the control group, and *n* = 15 for the MT treated group. In the GFAP analysis, *n* = 18 images were analyzed and averaged for both the control group and the MT treated group. In the Iba-1 and Caspase-3 analysis, *n* = 6 images were analyzed and averaged for both the control group and the MT treated group. In the NeuN analysis, *n* = 15 images were analyzed and averaged for the control group, and *n* = 14 for the MT treated group.

### 2.2. PCR Study

#### 2.2.1. Surgery

Twelve C57BL/6 male mice were randomly split into two control groups and one experimental group (*n* = 4). Throughout the study, animal experiments and housing were approved by and kept in accordance with University of Pittsburgh Institutional Animal Care and Use Committee standards. Mice from all groups received daily 100 μL intraperitoneal injections of either saline or MT in saline at 30 mg/kg three days preceding and seven days following probe implantation. Surgical procedures were performed as described above with the following alterations. One control group did not receive implants and was included to compare the response to untouched neural tissues. Melatonin and saline-treated groups received four electrodes implanted in the same hemisphere, placed in a square formation, 2 mm apart from adjacent probes to the left of the sagittal suture. Probes were sealed in place via Kwiksil and dental cement, and the incision site was closed and coated with a topical antibiotic. Ketofen injections (5 mg/kg) continued daily for three days after surgery for pain management.

After 7 days, animals were sacrificed via overdose of isoflurane, in which a tissue soaked with 100% isoflurane was placed in the induction box along with the animal. After confirming the animal was deceased, the brain was isolated, and 2 mm diameter biopsy punches were utilized to extract the necessary samples. Collections were taken from the area immediately surrounding each probe on the left implant side, and control samples from the right non-implant side of the brain.

#### 2.2.2. qPCR

Quantitative real-time polymerase chain reaction (qPCR) permits many target genes to be investigated quantitatively. Briefly, brain tissue was collected and stored at −80 °C until qPCR was performed. RNA extraction was performed using the Trizol method, and the concentration and purity of RNA samples were estimated by a spectrophotometer (NanoDrop, ThermoFisher, Waltham, MA, USA). cDNA was prepared using 100 ng of RNA and XLT cDNA SuperMix (Quantabio, Beverly, MA, USA). qPCR was run for each TNF-α and IL-6 gene primer in triplicate wells, along with GAPDH as a reference gene for each sample since GAPDH offered constant Ct values among control and implanted animals. Each well consists of a mixture of 12.5 μL of SYBR green Mix (Applied Biosystems, Waltham, MA, USA), 8 μL of nuclease-free water, 1 μL of cDNA, and 3.5 μL of a primer for each gene (Real-time PCR). All implanted animals were normalized to non-implanted control animals using the 2^(−ΔΔCt) method [37]. All samples were normalized with their respective Ct value of the GAPDH, to get ΔCt (ΔCt = Ct (target gene) − Ct (GAPDH)). For each gene, the ΔΔCt value was estimated by normalizing each implanted animal’s ΔCt value to all non-implanted control animals’ ΔCt value (ΔΔCt = ΔCt (implanted animal) − ΔCt (non-implanted animal). The relative fold-change was then estimated using the 2^(−ΔΔCt) method. One-way ANOVA with Bonferroni post hoc multiple comparison test was used for statistical analysis [38].

### 2.3. Two-Photon Microscopy

#### 2.3.1. Surgery

Eight transgenic mice expressing GFP with CX3CR1 promotor (The Jackson Laboratory, Bar Harbor, ME, USA) were randomly picked for the control (*n* = 4) or melatonin (*n* = 4) group. This animal strain has been used for tracking microglia in the brain and peripheral monocytes. Throughout the study, animal experiments and housing were approved by and kept in accordance with University of Pittsburgh Institutional Animal Care and Use Committee standards. The animal was anesthetized with Ketamine (22.5 mg/kg) and Xylazine (75 mg/kg) cocktail and then placed onto a stereotaxis (Narishige International USA, Amityville, NY, USA). After removing furs and skin tissue on the head, we placed a stainless-steel frame ((#CF-10, Narishige International USA, Amityville, NY, USA) onto the skull using dental cement (A-M Systems, Sequim, WA, USA). A 4 × 3 mm^2^ cranial window was created and a non-functional silicon-based planar electrode (NeuroNexus, Ann Arbor, MI, USA) was inserted at an angle of 30 degrees into the cortex at 200 μm/s for a total of 600 μm. Finally, the cranial window was sealed with silicone (Kwik-sil, World Precision Instruments) and a 3 mm square cover glass. Animals in the MT group were IP injected with 30 mg/kg melatonin daily for three days before surgery, and daily after that between 8 to 12 a.m. Animals in the control group were injected with the same volume of saline instead. Animals were also treated with 5 mg/kg Ketofen for 3 days post-surgery.

#### 2.3.2. Imaging

Two-photon images were acquired right after probe insertion for 2 h, and at 1 day, 2 days, 4 days, and 7 days post-surgery. The TPM setup has been previously described [39,40]. We used a tunable laser (Insight DS+; Spectra-Physics, Menlo Park, CA, USA) at 920 nm wavelength for excitation. The laser power was kept under 40 mW to avoid thermal damage to the tissue. Animals were IP injected with 0.05 cc 1 mg/mL sulforhodamine 101 before imaging for visualization of blood vessels.

Two-photon images focused on a 407.5 µm × 407.5 µm area around the implanted electrodes. Three-dimensional time-lapse two-photon microscopic images were acquired for 40 min after probe implantation at a speed of 1 min per 3D scan. Z-stack images were taken from the brain surface to the deepest imageable region at 2 h 1 day, 2 days, 4 days, and 7 days post-surgery.

#### 2.3.3. Image Analysis

Two-photon images were analyzed with ImageJ using previously developed methods [39,40,41]. Briefly, we manually tracked the microglial end-feet movement from 3D time-lapse videos. As for the microglial coverage quantification, we first rotated the stack to align it to the plane of the probe surface, and then threshold a 2D projected image from a 30 µm thick stack at the probe surface.

## 3. Results

### 3.1. Histology

**Figure 1:**
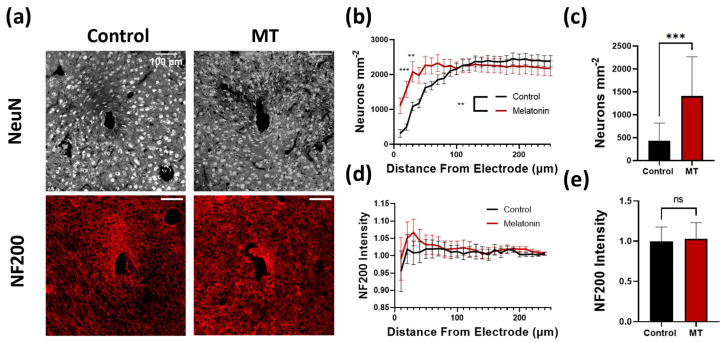
Melatonin (MT) administration preserves neuron density near the electrode site. (**a**) Representative images for NeuN (neurons) and NF200 (axons) staining in control and MT groups. (**b**) Plot of neuron body density (counted neurons per square μm) versus distance from implanted electrode center. (**c**) Bar graph depicting neuron density at 20 μm from electrode site. (**d**) NF200 intensity normalized to image corners versus distance from electrode. (**e**) Bar graph depicting normalized NF200 intensity at 20 μm from electrode site. (**: *p* < 0.01, ***: *p* < 0.001, two-way ANOVA).

Neural electrodes function by recording the electrical signals transmitted between neurons and are heavily dependent on the number of and distance to nearby neuronal cell bodies. Brain slices were stained with NeuN and NF200 to identify potential differences in neuronal counts as well as axon density, respectively, proximal to the electrode implant site (Figure 1 and Figure S1). Immediately surrounding the site, a significantly higher density of neurons was found adjacent to the implants in MT-treated animals compared to the untreated animals (*p* < 0.001 at 20 μm and *p* < 0.01 at 30 μm, two-way Anova) (Figure 1b). Furthermore, the number of neurons per square μm within the MT treated animals was nearly double that of the control group within 30 μm of the implant (Figure 1c). Moving radially from the site, neuron density remained higher in the tissues of MT-treated animals until roughly 100 μm away from the electrode, after which differences between the experimental and control groups were negligible. NF200 staining indicated no significant differences between groups, indicating a lack of variation in axonal density around the implants regardless of drug treatment (Figure 1d,e).

**Figure 2:**
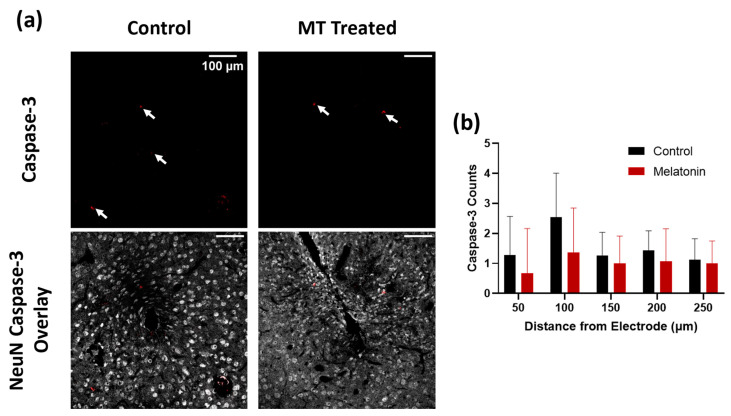
Melatonin (MT) decreases Caspase-3 expression in acute electrode implantation. (**a**) Representative images for Caspase-3 (apoptosis marker) staining in control and MT groups. White arrows indicate areas with Caspase-3 expression. Overlay with NeuN (grey) and Caspase-3 (red) to indicate neuron death. (**b**) Caspase-3 counts for both groups at different distances from electrode center (n.s., Two-way ANOVA).

Caspase-3 counts were used to confirm the health of neurons around electrodes in MT-treated animals as compared to controls (Figure 2a and Figure S1). Caspase-3 is a marker for apoptotic cell death, particularly neuron death in this case, and is a helpful indicator for the possible inflammation-induced decline in healthy neurons. Manual counts of Caspase-3 were generally lower in MT-treated animals than in control animals, especially within 100 μm from the implant site, though this difference was not statistically significant due to the very small number of caspase-3 positive cells (Figure 2b).

**Figure 3:**
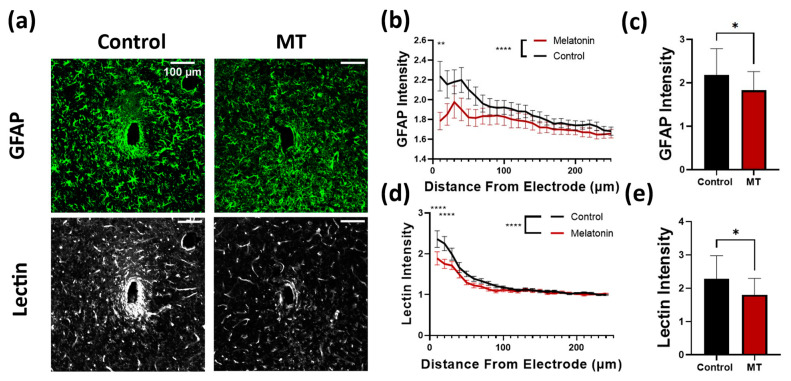
Melatonin (MT) administration prevents astrocytic activation near the electrode site. (**a**) Representative images for GFAP (astrocytes) and tomato lectin (vasculature and microglia) staining in control and MT groups. (**b**) Plot of GFAP intensity normalized to image corners versus distance from electrode center. (**c**) Bar graph depicting normalized GFAP intensity at 50 μm from electrode site. (**d**) Tomato lectin intensity normalized to image corners versus distance from electrode. (**e**) Bar graph depicting normalized tomato lectin intensity at 50 μm from electrode site. (*: *p* < 0.05, **: *p* < 0.01, ****: *p* < 0.0001, Two way ANOVA).

Next, we examined the extent of astrocytic and microglial encapsulation of the electrodes, as well as the extent of vascularization around the electrodes. GFAP staining was used to quantify reactive astrocytes, with tomato lectin serving as a secondary marker of microglia in addition to blood vessels (Figure 3a and Figure S2). Both stains revealed significantly greater intensity surrounding the implants in control animals compared to the MT-treated group. The difference in GFAP intensity was highest near the electrode site, dropping off as the radius increased (Figure 3b). Both MT treated and control tissues displayed significantly elevated reactive astrocyte expression relative to tissues far from the electrode, while MT treatment appears to have minimized this relative increase (Figure 3c). The same can be said for the tomato lectin staining, in which the difference between control and MT treated groups was greatest near the electrode and diminished outward (Figure 3d). Significant differences between the two groups at individual radii occurred within 20 μm of the implant site, and overall tomato lectin intensity was significantly greater for untreated animals (Figure 3e). The average intensity of tomato lectin surrounding the control implants peaked at more than double that of unaffected tissue, while MT-treated averages were below this threshold.

**Figure 4:**
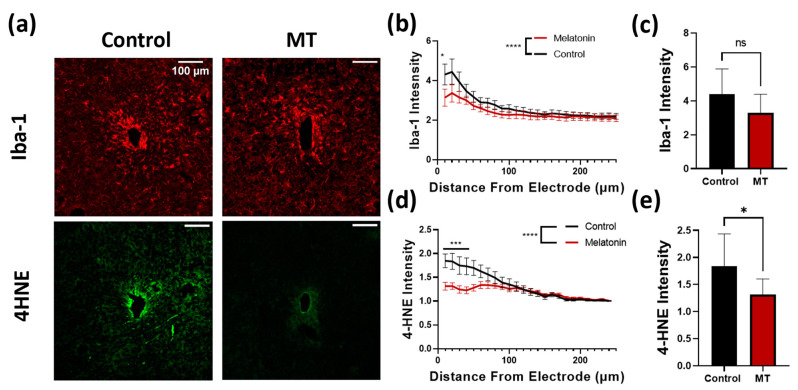
Melatonin (MT) administration prevents microglial activation and lipid peroxidation near the electrode site. (**a**) Representative images for Iba-1 (microglia) and 4HNE (lipid peroxidation) staining in control and MT groups. (**b**) Plot of Iba-1 intensity normalized to image corners versus distance from electrode center. (**c**) Bar graph depicting normalized Iba-1 intensity at 50 μm from electrode site. (**d**) 4HNE intensity normalized to image corners versus distance from electrode. (**e**) Bar graph depicting normalized 4HNE intensity at 50 μm from electrode site. (*: *p* < 0.05, ***: *p* < 0.001, ****: *p* < 0.0001, two-way ANOVA).

Following implantation, microglia are often the quickest cells to respond to the implantation. Upon activation, microglia increase the generation of ROS. These ROS serve as signaling molecules, increasing inflammatory cell recruitment. Further, ROS are damaging to both the electrode itself and the surrounding tissues. Iba-1 and 4HNE stains were performed to assess the extent of the inflammatory reaction and oxidative stress following implantation (Figure 4a). Iba-1 staining intensity was utilized to compare the microglia response to the implanted probes between groups. Both the number of cells as well as the expression of Iba-1 per cell increase as a result of the inflammatory response; therefore, intensity is used here to represent the degree of the reaction by summing the two [42]. For the MT-treated animals, the intensity of Iba-1 staining was markedly lower within a 10 μm radius of the implant, and overall, the population of microglia was significantly lower compared to the control group (Figure 4b,c). ROS generated by the microglia result in damage to the cell membranes of nearby cells. Lipid peroxidation, evaluated with 4HNE stain, was used to evaluate the extent of oxidative stress in the tissue caused by the implants (Figure 4d). 4HNE staining was significantly lower in the MT treated group than in the control group, with a notable difference being evident up to nearly a 100 μm radius (Figure 4e).

### 3.2. PCR

Tissues were collected from three groups as previously described and assessed for quantitative differences in IL-6 and TNF-α RNA expression. Both genes are used as indicators of activation of the inflammatory pathway within the brain tissue; expression relative to the housekeeping gene GADPH elucidates the magnitude of the inflammatory response occurring near the electrode sites.

**Figure 5:**
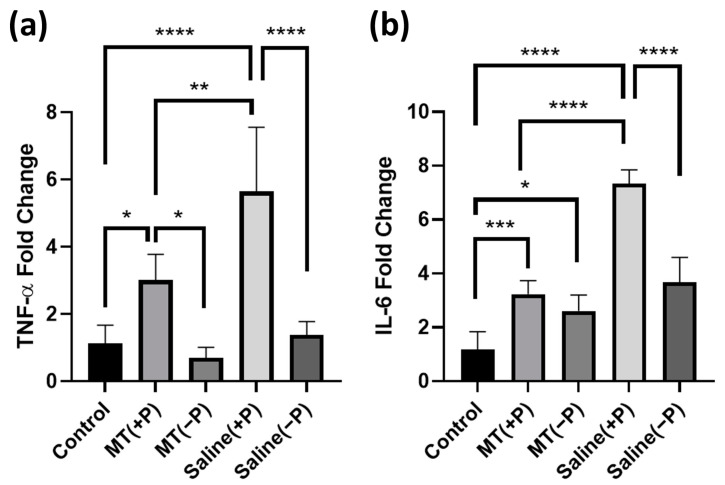
PCR relative quantification of TNF-α and IL-6-fold change indicative of inflammatory response under various conditions. Fold change is relative to assay standards. (**a**) Comparison of TNF-α fold change near electrode implant between groups. (**b**) Comparison of IL-6-fold change near electrode implant between groups. (*: *p* < 0.05, **: *p* < 0.01, ***: *p* < 0.001, ****: *p* < 0.0001. One way ANOVA). Groups: Control (untouched neural tissue), MT(+P) (MT-treated neural tissue with probe implanted), MT(−P) (MT-treated neural tissue without probe implanted), Saline(+P) (saline-treated neural tissue with probe implanted), and Saline(−P) (saline-treated neural tissue without probe implanted).

TNF-α fold change (calculated relative to the non-implanted control animals) indicated significant differences between the groups (Figure 5a). TNF-α expression significantly increased on the implanted compared to the contralateral non-implanted side, indicating an increase in inflammation in the brain tissue surrounding the probes (one-way ANOVA *p* < 0.05). Treatment with melatonin minimized this increase significantly, and TNF-α expression was reduced by 46.7% on the implanted side for MT-treated animals relative to probe implanted animals with no MT (one-way ANOVA *p* < 0.05).

Interleukin-6 (IL-6) exhibited a very similar trend (Figure 5b). Under the same experimental conditions, there was an increase in IL-6 expression on the probe implanted side versus the non-implanted side, though this increase was not significant for MT-treated animals compared to its contralateral side. In addition, average IL-6 expression decreased by 55.9% for the MT-treated animals as compared to the control implant group on the side with the probe implanted.

### 3.3. Two-Photon Microscopy

**Figure 6:**
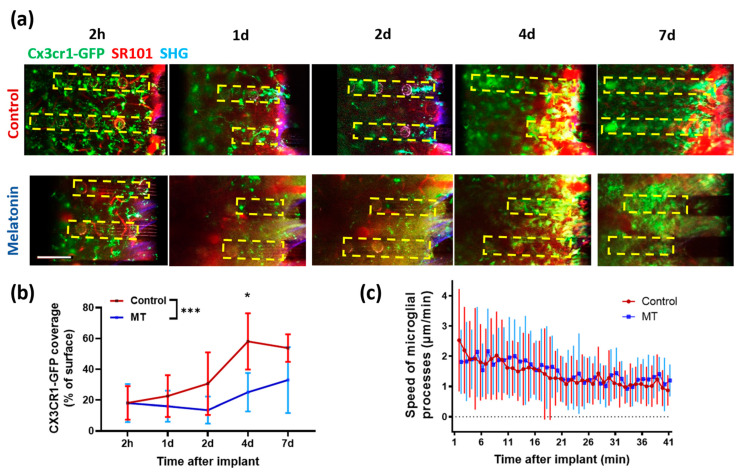
Chronic TPM imaging of dummy electrodes over time. (**a**) Representative TPM images showing the surface of electrodes over 7 days post insertion. Green, CX3CR1-GFP; red, blood vessel; blue, second-harmonic signal from collagen. Yellow dotted boxes indicate the location of electrodes. (**b**) Quantification of microglial coverage on the electrode surface over time. Mean ± SD are plotted. Two-way ANOVA followed by multiple comparisons at each time point (*: *p* < 0.05, ***: *p* < 0.001). (**c**) The movement speed of microglial end-feet after electrode implantation. Mean ± SD are plotted.

To investigate how and when melatonin begins affecting inflammatory responses toward neural probes, microglial activities were monitored after neural probe implantation in vivo using TPM in CX3CR1-GFP mice (Figure 6a). Microglia, the resident immune cells in the brain, have been reported as the first responder to neural electrode implantation and could trigger a series of inflammatory responses. Prior TPM works have revealed that microglial morphology is dynamically changing in the brain: in homeostatic conditions, microglia constantly survey the surrounding environment with moving processes [43]; upon neural electrode implantation, microglia first send processes toward the implant and then tend to encapsulate the device [40]. To illustrate whether MT influences microglial process extension and encapsulation, we tracked the speed of microglia processes moving toward the implant immediately after insertion of the neural probe (Figure 6c), as well as microglia coverage of the probe surface at 2 h, 1 day, 2 days, 4 days, and 7 days after probe implant (Figure 6b).

Control and MT-treated animals showed similar trends in the speed of microglial end-feet moving towards the implant. Microglial end feet in both groups moved immediately toward the implant upon probe insertion, and the speed decreased gradually and stabled at around 1 µm/min after about 30 min (Figure 6c), indicating that pre-implant MT treatment has minimum effect on the initial microglial processes’ extension. However, the microglial encapsulation of implants was different during our imaging period (Two-way ANOVA, *p* = 0.0001). Surface coverage was about the same at 2 h, but after day 1, the microglial coverage in control animals continuously increased while that in the MT treated group remained significantly lower. As shown in Figure 6a,b, the averaged microglial coverage of probe surface was lower on MT-treated animals compared to saline-injected animals from day 1 to day 7 post-implant, suggesting that MT treatment affects the microglia/macrophage recruitment to the implant surface. When comparing the microglial coverage data between MT and saline-treated animals on each day of imaging, day 4 post-implantation showed a statistically significant difference (*p* = 0.023), indicating the temporal dynamics of MT’s anti-inflammatory effects.

## 4. Discussion

Previous investigations have examined the effect of chronic melatonin administration on the long-term performance of neural recording electrodes. These investigations have demonstrated the therapeutic effects of melatonin, which decreased tissue inflammation and oxidative stress, and increased the recording lifetime of neural electrodes. It was also shown that MT not only exhibits anti-inflammatory but also anti-apoptotic properties, increasing the number of viable neurons near the electrode at 16 weeks post-implant. When chronically administered, MT acts to reduce overall host tissue response and states of increased inflammation [29,36,44,45,46].

While previous studies were useful to determine the effects of MT administration on recording performance, these studies did not investigate several acute aspects which are important for understanding how MT reduces trauma after the initial implantation injury [36]. This study aimed to address these loose ends and elucidate MT’s effects on the surrounding tissue a week after electrode insertion.

The histological analysis yielded results consistent with previous studies [36,47,48]. While neuron density diminished near the electrode site in untreated control animals at just one-week post-implant, administration of MT preserved this density significantly. This notion that MT prevents loss of neurons during acute insertion injury provides a biological explanation for the increased single-unit recording yield on day zero and day seven shown in our previous study [36].

Iba-1 and 4HNE, stains for microglia and lipid peroxidation, respectively, also validate MT’s known anti-inflammatory and antioxidative properties [44,49,50]. MT reduced the number of microglia surrounding the implants, as indicated by the significant decrease in Iba-1 staining as compared to the control tissue. Additionally, 4HNE staining results strongly suggest that MT reduces oxidative stress in tissue adjacent to the probes in that the staining is significantly lighter in MT-treated tissue. Antioxidants, such as resveratrol, are well known for reducing tissue damage after neural probe insertion, as well as attenuating microglia/macrophage response to the implant site [17,18]. Similar to resveratrol, MT’s antioxidative effects can be employed to reduce the oxidative stress resulting from implantation. MT acts as a direct free radical scavenger, while also regulating the gene expression of several antioxidative enzymes naturally produced by the target tissue [30,38]. Antioxidative enzymes such as superoxide dismutase as well as glutathione peroxidase are upregulated in response to MT while downregulating enzymes such as nitric oxide synthase [51,52].

Furthermore, MT’s anti-inflammatory properties are confirmed by the trends in GFAP staining. The expression of this reactive astrocyte marker was markedly reduced surrounding the electrodes implanted in the MT group. Tomato lectin staining, which is a secondary marker for microglia, confirms this finding. MT is known well to exhibit anti-inflammatory properties and shows this same outcome in the treated tissue surrounding the implanted electrodes [53,54,55].

Results from the PCR experiment further highlighted MT’s anti-inflammatory properties alongside histology findings. Implantation trauma causes a release of cytokines via the NF-KB pathway, signaling nearby cells to mount an inflammatory response to the inserted probe. Activation of this pathway also leads to significantly increased downstream TNF-α and IL-6 gene expression in affected tissue [56,57]. MT’s anti-inflammatory action attenuates this response, minimizing the activation of the NF-KB pathway and thus preventing the production of TNF-α and IL-6 [58,59]. In the case of both genes (TNF-α and IL-6), the MT treated probe implant side was more similar to the control non-implant expression than to the control implant probe site expression. This indicates that MT was effective in maintaining normal levels of inflammatory pathway expression, even with significant implant trauma induced.

TPM excels at in vivo sub-cellular resolution imaging. In the context of the neural-electrode interface, previous TPM studies suggest that microglia are an important first-line responder and extend processes towards the implanted device immediately after insertion [40,41]. Twelve hours after implantation, microglia become ameboid in shape, and blood-borne monocytes begin adhering and accumulating on the device surface. Here, we use TPM to visualize how MT influences the inflammatory responses in vivo in CX3CR1-GFP mice which labels both microglia in the brain parenchyma and peripheral macrophages. To determine if MT administration affects the cellular machineries supporting process extension, we measured the speed of the microglia process movement towards the implant. The results show that MT does not affect the process extensions of microglia. However, from day 1 to day 7 post-implantation, the CX3CR1-GFP coverage of the implanted electrodes was significantly reduced in the MT group, indicating that MT has an influence on later phases of inflammatory responses, such as microglial ameboid transition, phagocytosis, and recruitment of peripheral macrophage. These findings align with our histological and PCR results, where the density of Iba-1 labeled microglia was remarkably lower and the pro-inflammatory genes expression was significantly reduced in MT-treated animals compared to control animals. It has been shown that microglia/macrophage polarization is dynamically changing post-brain injury [60]. It would be interesting to differentiate the polarization state of microglia in future studies.

Admittedly, this study only provides a snapshot of immunohistochemical, molecular, and real-time morphological analysis of the cellular reaction to MT administration during an acute phase of implantation [61,62]. There is potential to expand the time points of the study as well as extend the depth for chronic in vivo imaging around neural implants with three-photon technologies [63], transparent electrode arrays [64,65], or with extra intracortical optical devices [66,67].

While systemic administration of melatonin is effective in modulating the tissue response to neural electrodes, it may bring unwanted side effects. Future work will focus on the controlled release of melatonin and other similar molecules from the surface of chemically modified electrodes to locally reduce inflammation without the need for a systemic injection.

## 5. Conclusions

While our previous study confirmed melatonin’s success in mitigating chronic recording and tissue degradation, in this study, we assert that melatonin exhibits neuroprotective qualities as early as one week after implantation [40]. Our TPM and immunohistological studies show that melatonin inhibits microglial encapsulation and astrocytic activation and preserves neuron density among other benefits in the acute setting. Further PCR analysis revealed that melatonin significantly decreases the expression of inflammatory markers TNF-α and IL-6 and, thus, the inflammatory response to the inserted probes.

## Data Availability

All data produced in this study are available upon reasonable request from the corresponding author.

## Supplementary Materials

The following supporting information can be downloaded at: https://www.mdpi.com/article/10.3390/antiox11081628/s1, Figure S1: Zoomed and binned representative images for NeuN and NF200 from main document for reference. Bins (red concentric ovals) are 10 μm wide and each image contains 25 bins total. Center bin is marked with an x to indicate that it is not considered in the intensity analysis; Figure S2: Zoomed and binned representative images for GFAP and tomato lectin from main document for reference. Bins (red concentric ovals) are 10 μm wide and each image contains 25 bins total. Center bin is marked with an x to indicate that it is not considered in the intensity analysis.
