# Supplementary material for: Melatonin Decreases Acute Inflammatory Response to Neural Probe Insertion
Source: Antioxidants (Basel). 2022 Aug 22;11(8):1628. doi: 10.3390/antiox11081628 (PMC9405074; doi:10.3390/antiox11081628)
Supplement: Supplementary file 1 [file antioxidants-11-01628-s001.zip › antioxidants-1841707-supplementary.pdf]

Supplementary Materials

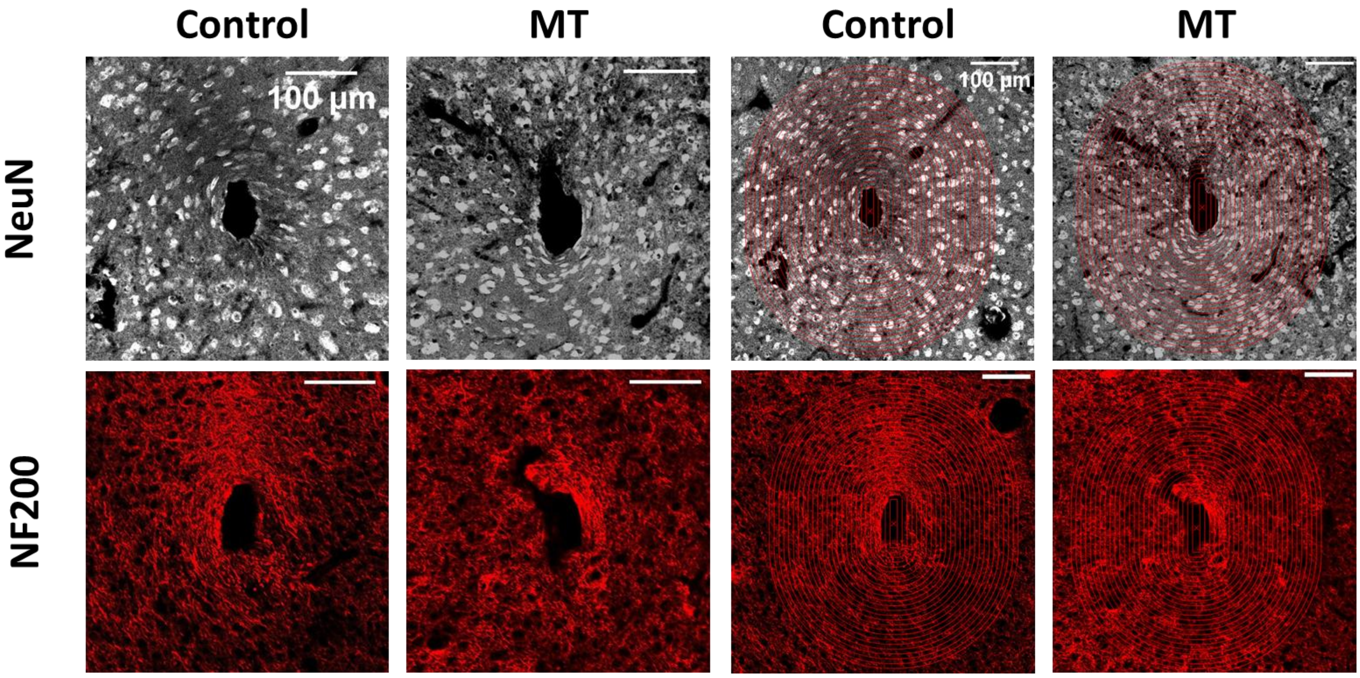

Figure S1. Zoomed and binned representative images for NeuN and NF200 from main document for reference. Bins (red concentric ovals) are 10 µm wide and each image contains 25 bins total. Center bin is marked with an x to indicate that it is not considered in the intensity analysis.

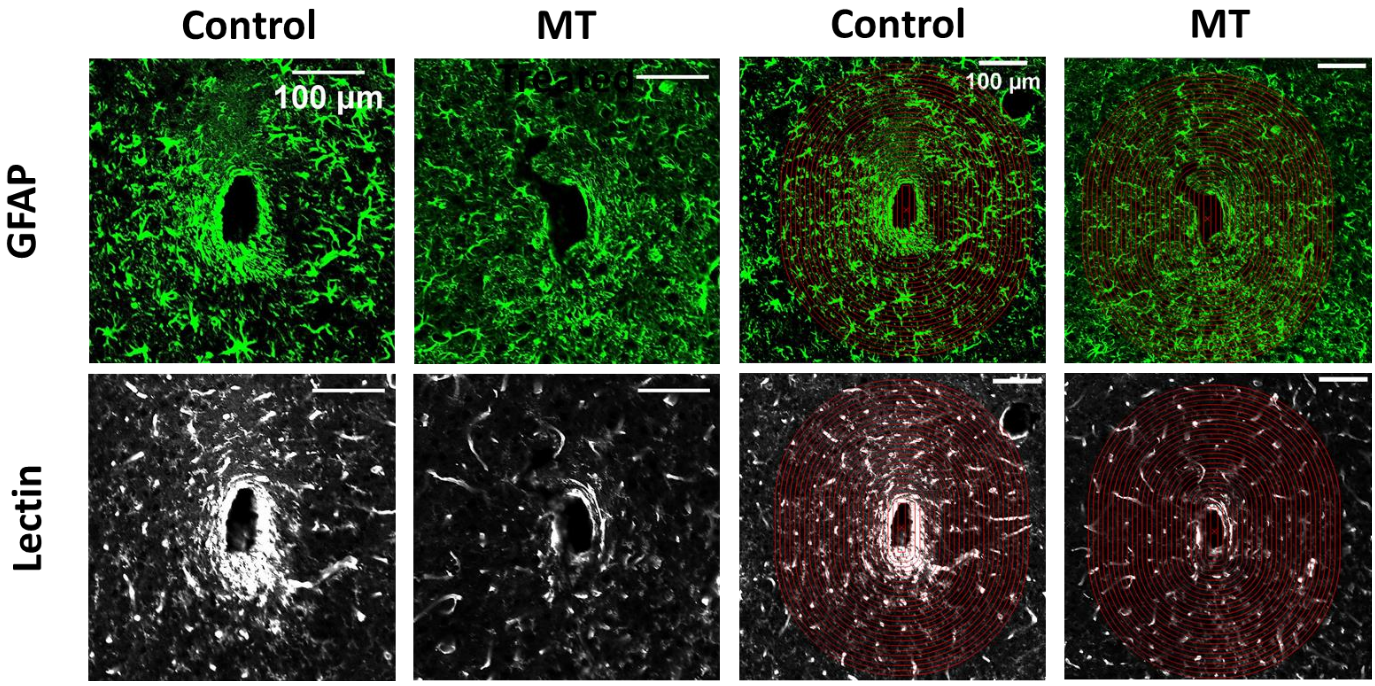

Figure S2. Zoomed and binned representative images for GFAP and tomato lectin from main document for reference. Bins (red concentric ovals) are 10 µm wide and each image contains 25 bins total. Center bin is marked with an x to indicate that it is not considered in the intensity analysis.
